# Genetic Landscape of *SH3TC2* variants in Russian patients with Charcot–Marie–Tooth disease

**DOI:** 10.3389/fgene.2024.1381915

**Published:** 2024-06-06

**Authors:** Olga Shchagina, Aysylu Murtazina, Polina Chausova, Mariya Orlova, Elena Dadali, Sergei Kurbatov, Sergey Kutsev, Aleksander Polyakov

**Affiliations:** ^1^ Research Centre for Medical Genetics, Moscow, Russia; ^2^ Research Institute of Experimental Biology and Medicine, Voronezh State Medical University named After N.N. Burdenko, Voronezh, Russia; ^3^ Saratov State Medical University, Saratov, Russia

**Keywords:** SH3TC2, CMT4C, mutation, neuropathy, Charcot-Marie-Tooth disease type 4C, hereditary motor and sensory neuropathy, HMSN

## Abstract

**Introduction:**

Charcot–Marie–Tooth disease type 4C (CMT4C) OMIM#601596 stands out as one of the most prevalent forms of recessive motor sensory neuropathy worldwide. This disorder results from biallelic pathogenic variants in the *SH3TC2* gene.

**Methods:**

Within a cohort comprising 700 unrelated Russian patients diagnosed with Charcot–Marie–Tooth disease, we conducted a gene panel analysis encompassing 21 genes associated with hereditary neuropathies. Among the cohort, 394 individuals exhibited demyelinating motor and sensory neuropathy.

**Results and discussion:**

Notably, 10 cases of CMT4C were identified within this cohort. The prevalence of CMT4C among Russian demyelinating CMT patients lacking the *PMP22* duplication is estimated at 2.5%, significantly differing from observations in European populations. In total, 4 novel and 9 previously reported variants in the *SH3TC2* gene were identified. No accumulation of a major variant was detected. Three previously reported variants, c.2860C>T p. (Arg954*), p. (Arg658Cys) and c.279G>A p. (Lys93Lys), recurrently detected in unrelated families. Nucleotide alteration p. (Arg954*) is present in most of our patients (30%).

## 1 Introduction

Biallelic pathogenic variants in the *SH3TC2* gene cause autosomal recessive demyelinating neuropathy (Charcot-Marie-Tooth disease, CMT) type 4C (CMT4C) OMIM#601596. According to the new classification proposed in 2015, this form of neuropathy is called AR-CMTde-SH3TC2 (Mathis et al., 2015). This type of CMT is characterized not only by peripheral neuropathy but also by early-onset spinal deformities and occasional cranial nerve damage that may be manifested by hearing loss (Senderek et al., 2003; Lerat et al., 2019b). The SH3TC2 protein contains the Src homology-3 (SH3) domains and tetratricopeptide repeat (TPR) motifs, localized in the plasma membrane of Schwann cells of peripheral nerves (Arnaud et al., 2009). The SH3TC2 protein is an effector for the small GTP-binding protein Rab11, which plays a key role in regulating endosome recycling processes. Mutations that change the amino acid sequence of SH3TC2 can disrupt this interaction with Rab11. Rab11 also plays a role in the regulation of myelination by Schwann cells in culture (Stendel et al., 2010).

In recent years, novel therapeutic strategies have emerged for addressing hereditary disorders, including advancements in gene replacement therapy, antisense oligonucleotides, and other innovative approaches (Eggermann et al., 2020; Wang et al., 2020). Significant progress has been made in the development of medications targeting hereditary neuropathy and other monogenic diseases (Gautier et al., 2021; Pisciotta et al., 2021; Ozes et al., 2022). Notably, gene replacement therapy holds particular significance in the context of recessive neuropathy, where the loss of gene function is implicated. This therapeutic approach has demonstrated notable efficacy, as evidenced by improvements in the phenotype of mice with CMT4C (Schiza et al., 2019).

Here we present the genotypic profiles and clinical characteristics of individuals harboring biallelic *SH3TC2* variants from Russia.

## 2 Materials and methods

The cohort under investigation comprised 700 non-related probands who sought consultation at the Research Centre for Medical Genetics between January 2017 and December 2020. In the diagnostic evaluation, CMT was considered as a potential differential diagnosis for all patients. A clinical diagnosis was established through a comprehensive assessment encompassing both clinical and electrophysiological examinations. The primary diagnostic criteria for hereditary peripheral neuropathies included the presence of a steppage gait, atrophy of hand, lower leg, and foot muscles, deformities in the hands and feet, as well as a reduction or absence of tendon reflexes. Sensitivity disorders were also noted in some cases.

Among the cohort, 394 individuals exhibited demyelinating motor and sensory neuropathy, 236 demonstrated axonal motor and sensory neuropathy, 22 were diagnosed with hereditary motor neuropathy, 3 exhibited sensory neuropathies, and 44 presented with hereditary neuropathy with liability to pressure palsies. Nerve conduction study results were available for all probands included in the study.

DNA was extracted from whole blood samples using a Wizard^®^ Genomic DNA Purification Kit (Promega, United States) according to the manufacturer’s protocol. Quantitative analysis was carried out using the SALSA MLPA Probemix P405 (MRC-Holland). The probands’ DNA was analyzed using a custom AmpliSeq™ panel on an Ion Torrent S5 next-generation sequencer. The panel included coding gene sequences for the following genes: *AARS1, BSCL2, EGR2, FIG4, GDAP1, GJB1, HINT1, HSPB1, INF2, LITAF, LRSAM1, MFN2, MME, MORC2, MPZ, NDRG1, NEFL, PMP22, PRX, SH3TC2, SORD.* Sequencing results were processed using the standard automated algorithm for data analysis (Supplementary Table S1). The average coverage for all samples was 80 × with the coverage width of (20×) ≥ 90%–94%. The detected variants were called according to the nomenclature presented on the http://varnomen.hgvs.org/recommendations/DNA website.

To assess the population frequencies of the identified variants, we used samples from the “1000 Genomes” project, ESP6500, and The Genome Aggregation Database v2.1.1. To evaluate the clinical significance of the identified variants, we used the OMIM and CliVar databases and the HGMD^®^ Professional database v2021.3. VarSome (Kopanos et al., 2019) and Franklin (https://franklin.genoox.com—Franklin by Genoox) tools were used to assess the pathogenicity of variants. These tools allow simultaneous use of several predictors of pathogenicity within a single search query. The following programs were used to predict the splicing effect: SliceAI (Jaganathan et al., 2019), Pangolin (Zeng and Li, 2022), SPiP (Leman et al., 2022), Human Splicing Finder (Desmet et al., 2009) and NetGene2 (Hebsgaard et al., 1996). Assessment of the pathogenicity and causatively of genetic variants was carried out in accordance with the international recommendations for interpretation of data obtained by massive parallel sequencing.

Automated Sanger sequencing was carried out using an ABIPrism 3500xl Genetic Analyzer (Applied Biosystems, Foster City, CA, United States) according to the manufacturer’s protocol. Primer sequences were chosen according to the NM_024577.4 reference sequence. Sanger sequencing was used as a reference method by us to confirm the identified variants and to search for variants in the proband’s family members.

## 3 Results

Among the 700 non-related patients referred to our laboratory with a diagnosis of “peripheral neuropathy,” a subset of ten individuals exhibited biallelic pathogenic and likely-pathogenic variants in the *SH3TC2* gene. As anticipated, the manifestation of CMT4C was identified within the subset of patients exhibiting demyelinating neuropathy, consisting of 394 individuals. Consequently, the prevalence of CMT4C among Russian demyelinating CMT patients lacking the *PMP22* duplication was calculated to be 2.5%. Within this cohort, the overall count of affected individuals totaled 10, comprising seven isolated cases and three familial cases. For all SH3TC2 patients, the place of birth coincides with the place of residence. All but one of the families reported that they were Russian. One patient (1907) is a descendant of the interethnic union. His father is Udmurt by nationality and his mother is Russian. The distribution of clinical and electrophysiological data for these patients and information about the places of residence is detailed in Table 1.

**TABLE 1:** The *SH3TC2* gene variants spectrum and clinical features of patients with CMT4C. Y. - years; N.med. - median nerve; PAT - pathogenic variant (according to ACMG criteria); LPAT - likely pathogenic variant (according to ACMG criteria).

Patients ID	Place of residence	Affected family members	Age of onset, years	NCV (*n.med.,* m/s)	Peculiar clinical features	*SH3TC2* (NM_024577.4)	
						Variant 1	Variant 2
2711	Amur region	1	6	23	weakness of the facial muscles, wheelchair-dependent since 14 years	c.2860C>T	c.2860C>T
						(p. (Arg954*))	(p. (Arg954*))
						PAT (Lerat et al., 2019a)	PAT (Lerat et al., 2019a)
2718	Moscow region	1	1	23	S-shaped thoracolumbar scoliosis since the age of 15 years	c.3054–2A>G	c.1252C>T (p. (Gln418*))
						LPAT (Lupski et al., 2010)	LPAT (PM2, PVS1)
2893.1	Moscow	2 (an affected brother has the same variants)	36	31	scoliosis, congenital mild left ptosis	c.416T>C (p. (Leu139Pro))	c.1972C>T (p. (Arg658Cys)) PAT (Lupo et al., 2009)
						LPAT (PM2, PP3, PP1-M, PM3)	
3183	Saratov	1	2	28.8	n/a	c.2551_2554dup (p. (Val852Alafs*24))	c.416T>C (p. (Leu139Pro))
						PAT (Volodarsky et al., 2021)	LPAT (PM2, PP3, PP1-M, PM3)
3330.1	Moscow	1	5	22.6	n/a	c.2860C>T (p. (Arg954*))	c.3341del (p. (Pro1114Leufs*2))
						PAT (Lerat et al., 2019a)	PAT (Senderek et al., 2003)
3387	Bryansk region	1	11	39	n/a	c.279G>A; (p. (Lys93Lys)) (splice)	c.3157dup (p. (Leu1053Profs*36))
						LPAT (Laššuthová et al., 2011)	LPAT (PM2, PVS1)
3658	Moscow	1	10	32	thoracic scoliosis since the age of 2 years	c.2710C>T (p. (Arg904*))	c.2710C>T (p. (Arg904*))
						PAT (Senderek et al., 2003)	PAT (Senderek et al., 2003)
1907	Udmurt Republic	2 (an affected brother has the same variants)	14	n/a	severe kyphoscoliosis since the age of 4 y, restless tongue	c.530-1G>A	c.2491_2492del
						PAT (PM2, PVS1, PP5)	(p. (Leu832Hisfs*8))
						Yger et al. (2012)	PAT (Senderek et al., 2003)
3514	Moscow region	2 (an affected sis* has the same variants)	26	22.7	kyphoscoliosis	c.1177 + 5G>A	c.279G>A; (p. (Lys93Lys) (splice)) LPAT (Laššuthová et al., 2011)
						LPAT (Shchagina et al., 2023)	
3930	Moscow	1	10	32	n/a	c.2860C>T (p. (Arg954Ter))	c.416T>C (p. (Leu139Pro))
						PAT (Varley et al., 2015)	LPAT (PM2, PP3, PP1-M, PM3)

In the majority of cases (8 out of 10), the disease was caused by compound heterozygous variants. Notably, only two probands had previously reported pathogenic nonsense variants in a homozygous state: c.2710C>T (p. (Arg904*)) (Varley et al., 2015)—ClinVar: [VCV000021696.22] - and the most common pathogenic *SH3TC2* variant c.2860C>T (p. (Arg954*))—ClinVar: [VCV000002482.79]. The latter variant was detected in two additional families in a compound heterozygous state with other variants. In one proband, it co-occurred with a previously described deletion c.3341del (p. (Pro1114Leufs*2)) (Senderek et al., 2003)—ClinVar: [VCV000021699.4], and with a novel missense variant, c.416T>C (p. (Leu139Pro)) ClinVar: [SCV005042994], in another family. This missense variant was not present in the population databases such as gnomAD (https://gnomad.broadinstitute.org/), pathogenicity prediction programs evaluate it to be pathogenic (MetaRNN = 0.877 according to VarSome (Kopanos et al., 2019)). Furthermore, this particular novel variant was identified in two unrelated Russian families. In two brothers with late onset and mild disease courses, it was detected in the compound heterozygous state with a well-documented pathogenic missense variant c.1972C>T (p. (Arg658Cys)) ClinVar: [VCV000021690.42], previously reported in Slavic patients (Laššuthová et al., 2011). In another unrelated proband, the novel variant was detected with variant c.2551_2554dup (p. (Val852Alafs*24)) (Volodarsky et al., 2021) ClinVar: [VCV000916842.3].

A synonymous variant c.279G>A ClinVar: [VCV000216120.16] was detected in two non-related Russian families. It has previously been described in two patients from the Czech Republic in a compound heterozygous state with p. (Arg954*) and p. (Tyr169His) respectively (Laššuthová et al., 2011). Subsequent functional analysis conducted on this synonymous variant in 2012 demonstrated its effect on splicing (Laššuthová et al., 2012). In the current study, this variant was observed in two Russian families in a compound heterozygous state. In one family with two affected siblings the variant co-occurred with the variant c.1177 + 5G>A (Shchagina et al., 2023) ClinVar: [VCV000575267.10]. In another unrelated proband, this variant was found in a compound heterozygous state with a novel single-nucleotide duplication c.3157dup (p. (Leu1053Profs*36)). Duplication of the c.3157 nucleotide leads to the formation of a premature stop codon, which results in a probable loss of function. In one family, the disease Two previously reported pathogenic variants c.530-1G>A ClinVar: [VCV000637864.1] and c.2491_2492del (p. (Leu832Hisfs*8)) ClinVar: [VCV000021694.4] (Senderek et al., 2003) were found in one family with two affected siblings. Novel likely pathogenic variants, c.1252C>T (p. (Gln418*)) ClinVar: [SCV005042538] and c.3054–2A>G (Lupski et al., 2010) ClinVar: [VCV002736812.1], were detected in one proband. For the *SH3TC2*(NM_024577.4) c.3054-2A>G variant, the SpliceAI (Jaganathan et al., 2019) program predicts a probability of loss of the canonical acceptor splice site with a Δ score of 0.99, and activation of an acceptor site nine bases before the start of exon (Δ score 0,92). This change would lead to an extension of the exon 13 by three amino acids: p.Ser958MetinsCysSerGly. It will lead to a change in the structure of the TPR-like domain, the functions of which are not fully understood. Other prediction programs such as Pangolin (Zeng and Li, 2022), SPiP (Leman et al., 2022), Human Splicing Finder (Desmet et al., 2009) and NetGene2 (Hebsgaard et al., 1996) confirm the undoubted effect of this substitution on the acceptor site of exon 13 splicing. The variant was previously described in patients in a cohort study, but its pathogenicity was not determined (Lupski et al., 2010).

All examined subjects, except for two probands, had the disease onset during childhood, and all of them had either biallelic loss-of-function variants or combination of loss-of-function and missense variants. Two unrelated probands (cases 2893.1 and 3514) had late onset at the ages of 36 and 26 years, respectively. In one family (2893), the disease was caused by two missense variants, with one of them previously reported to be associated with late manifestation (Laššuthová et al., 2011). In another family (3514), the disease was caused by variants affecting splicing. c.1177 + 5G>A variant leads to the total absence of the normal transcript (Shchagina et al., 2023). The c.279G>A variant has previously been shown to affect splicing, leading to the insertion of 19 nucleotides from intron 3 with the formation of a premature stop codon TAA in position 127 in the amino acid sequence (Laššuthová et al., 2012). The clinical phenotype observed in patients with late disease onset aligns with CMT4C, exhibiting spinal deformities. While the majority of studies document the manifestation of this type of peripheral neuropathy in the first decade, there exist reports detailing similar late-onset presentations attributed to the milder effects of the mutations (Colomer et al., 2006). The data on clinical features is summarized in Table 2.

**TABLE 2:** Clinical features of patients with biallelic variants in the *SH3TC2* gene.

Clinical characteristics	Median (range)
Age of manifestation, years	8 (1–36)
	First decade (0–9 years): 4 patients
	Second decade (10-19): 4 patients
	Adult: 2 patients
*n.med.* NCV, m/s	29.9 (22.6–39)
Had scoliosis prior to atrophy (% of patients)	50%
Facial muscle weakness (% of patients)	20%

Severe scoliosis was observed in individuals from five unrelated families. In probands from three unrelated families, clinical features of peripheral neuropathy became apparent 8–10 years after the onset of spinal deformities. Within our cohort, two unrelated patients exhibited facial muscle weakness resulting from facial nerve damage. None of the patients or their siblings reported hearing loss. We found information about the examinations by an otolaryngologist in the medical records for six patients. The doctors did not mention hearing loss in their reports. Four out of ten patients in the chart with CMT4C have locked clinical information, making it difficult to establish genotype-phenotype correlations and conduct statistically significant analyses.

### 3.1 Discussion

The spectrum of detected pathogenic *SH3TC2* variants was quite diverse in comparison to other autosomal recessive neuropathies (Shagina et al., 2010; Shchagina et al., 2020), showing only an insignificant accumulation of the variant c.2860C>T (p. (Arg954*)). Along with that, three variants were detected in several non-related families. The most common nonsense variant is c.2860C>T (p. (Arg954*)) identified in 20% of all affected alleles. This variant is prevalent in most European populations and often occurs in Romani People (Colomer et al., 2006). Two variants c.1972C>T (p. (Arg658Cys)) and c.279G>A were previously described in Czech patients, possibly indicating Slavic origin, but these variants have also been described in non-Slavic populations (Capalbo et al., 2019). Three unrelated Russian families had a novel c.416T>C (p. (Leu139Pro)) missense variant in a compound heterozygous state with other variants. This variant affects the N-terminal region of the SH3TC2 protein and is located outside of the functional domains. This could be the reason for its relatively mild impact on the phenotype. Two other previously unreported variants c.3157dup (p. (Leu1053Profs*36)) and c.1252C>T (p. (Gln418*)) lead to more dramatic consequences due to the shift in the reading frame.

The calculations conducted in this study revealed that the prevalence of CMT4C among Russian demyelinating CMT patients, who do not exhibit the *PMP22* duplication, is estimated to be 2.5%. Notably, this prevalence rate differs significantly from that observed in European populations.

The prevalence of CMT4C varies across populations. In individuals with demyelinating hereditary peripheral neuropathy lacking the *PMP22* duplication, CMT4C stands out as the prevailing autosomal recessive subtype in certain countries, constituting over 20% in Italy (Piscosquito et al., 2016) and the Czech Republic (Laššuthová et al., 2011), 26% in Greece (Kontogeorgiou et al., 2019). In India at 9% (Nagappa et al., 2023), in Germany at 4.9% (Rudnik-Schöneborn et al., 2016) and even rarer in Japan, accounting for only 1.76% (Yuan et al., 2018) and in China at 1.46% (Sun et al., 2022) among all patients with myelinopathy.

It should be noted that it is not always possible to directly compare the frequency of a particular type of neuropathy in different countries. Many studies have been conducted using fundamentally different patient groups. Some authors calculated the contribution of Charcot-Marie-Toute disease 4C (CMT4C), taking into account all patients with CMT (Nagappa et al., 2023), all CMT1 patients (Rudnik-Schöneborn et al., 2016; Yuan et al., 2018; Sun et al., 2022), while other studies selected patients with CMT1 who do not have a duplication of the PMP22 gene (Laššuthová et al., 2011; Piscosquito et al., 2016; Kontogeorgiou et al., 2019). In addition, patients with both parallel and heterozygous mutations are accounted for differently in different studies.

Large deletions have been described in the *SH3TC2* gene (Cortese et al., 2020; Pyromali et al., 2022; Rehbein et al., 2023). In all cases, targeted searches for long deletions were carried out using various methods, including analysis of panel sequencing data and long-range PCR for target patients with specific clinical features of demyelinating polyneuropathies and one pathogenic variant in the *SH3TC2* gene. This search strategy is due to the large number of false positive and negative results in the analysis of amplicon panel data for the detection of extended mutations, as well as the complexity and high cost associated with long-range PCR methods. Among 394 patients with demyelinating polyneuropathy in the study group, none had heterozygous pathogenic variants of the *SH3TC2* gene. This confirms the low incidence of CMT4C (Charcot-Marie Tooth disease type 4C) in Russia. Given the contribution of large deletions to the mutation structure of the *SH3TC2* gene, as reported by the HGMD database, it is possible that we may have missed one patient with homozygous or compound heterozygous deletions in our study cohort. This finding should be taken into consideration when assessing the role of CMT4C in the development of myelinopathy in Russia.

The age of disease onset exhibited significant variability, spanning from the first year of life to 36 years within our cohort. In some cases, individuals with CMT4C experienced a gradual progression of the disease, maintaining ambulatory movement. Conversely, one patient became wheelchair-dependent at the age of 14. Such heterogeneity in symptom severity is a hallmark of CMT4C (Senderek et al., 2003). Remarkably, within two families, the manifestation of the disease occurred at a later age, specifically after 20 years. The delayed onset and relatively mild progression of the disease are distinctly linked to the presence of specific pathogenic variants. Notably, missense and non-canonical splice site variants were identified as variants enabling the preservation of some activity of the SH3TC2 protein.

It is noteworthy that the clinical features associated with CMT4C, as described, including early-onset scoliosis and cranial nerve damage leading to facial muscle weakness (Azzedine and Salih, 1993; Houlden et al., 2009), were observed in only 50% and 20% of the examined patients, respectively. In three probands, spinal deformities manifested 8–10 years prior to the onset of peripheral neuropathy, a distinctive clinical characteristic of CMT4C. Notably, Senderek et al., 2003 and other researchers have observed that, in some cases, scoliosis is presented as the primary complaint (Azzedine and Salih, 1993). We have no information about hearing disorders. It should be noted that the limitation of the presented clinical analysis may be the lack of detailed clinical information about some patients.

The variability observed in clinical pictures of individuals with *SH3TC2* variants may be attributed to the accumulation of distinct pathogenic variants across diverse populations. However, it is essential to acknowledge that other factors influencing the progression of hereditary peripheral neuropathies cannot be disregarded.

## Data Availability

The original contributions presented in the study are included in the article/Supplementary Material, further inquiries can be directed to the corresponding author.
